# Intentional Versus Incidental Encoding of Emotional and Navigational Memory: Preliminary Data in Healthy Young Adults

**DOI:** 10.3390/brainsci16101113

**Published:** 2026-10-15

**Authors:** Andrea Di Piero, Alessia Bonavita, Laura Piccardi, Maria Casagrande, Cecilia Guariglia

**Affiliations:** 1 Department of Psychology, Sapienza University of Rome, 00185 Rome, Italy; andrea.dipiero@uniroma1.it (A.D.P.); cecilia.guariglia@uniroma1.it (C.G.); 2 Faculty of Law, Giustino Fortunato University, 82100 Benevento, Italy; a.bonavita@unifortunato.eu; 3 IRCCS San Raffaele Cassino Hospital, 03043 Cassino, Italy; 4 Department of Dynamic and Clinical Psychology, Sapienza University of Rome, 00185 Rome, Italy; maria.casagrande@uniroma1.it; 5 Cognitive and Motor Rehabilitation and Neuroimaging Unit, IRCCS Fondazione Santa Lucia, 00179 Rome, Italy; 6 Department of Medicine and Health Sciences, University of Molise, 86100 Campobasso, Italy

**Keywords:** episodic memory, navigation, incidental memory, emotional landmarks, landmark-based navigation, familiarity

## Abstract

**Highlights:**

**What are the main findings?**

- Intentional encoding instructions selectively enhanced episodic memory for negative and neutral emotional images, whereas positive images were remembered equally well regardless of instructions.
- Navigational memory (routes and landmarks) did not benefit from encoding instructions, showing that the intentional–incidental distinction is valence- and domain-specific rather than general.

**What are the implications of the main findings?**

- Positive emotional content appears to be well remembered under incidental encoding, a property that could be exploited to design rehabilitation and clinical memory assessment tools that do not rely on effortful, intentional encoding.
- These pilot effect-size estimates lay the groundwork for an adequately powered study extending this paradigm to older adults and individuals at risk of pathological aging, in whom incidental memory processes may remain relatively preserved.

**Abstract:**

Background/Objectives: episodic memory refers to the ability to place events within a specific spatial and temporal context, making its association with navigation unsurprising. Incidental episodic memory involves encoding and retrieving information without explicit instruction, a common process in everyday life whose sensitivity to emotional valence warrants further investigation. Methods: sixty-three college students (mean age = 25 years; 28 females) completed two versions of the same task, differing only in the instructions given: one directed attention to emotionally charged images encountered along a route through a city environment; the other directed attention to the route itself. Results: Mann–Whitney tests on uncontaminated first-session conditions and linear mixed models all indicated an advantage of intentional over incidental encoding for the spatial localization of emotional images. The advantage was valence-selective, present for negative and neutral stimuli but absent for positive ones. Signal detection analyses indicated that intentional instructions selectively increased sensitivity (d′). The response criterion also shifted, but in opposite directions for negative and neutral stimuli, so the sensitivity gain cannot be attributed to a uniform change in response bias. The effect was domain-specific: sensitivity for the spatial localization of emotional images increased, whereas sensitivity to navigational route did not improve. Conclusions: positive material thus appears to be encoded spontaneously, with potential implications for rehabilitation and clinical assessment.

## 1. Introduction

The relationship between episodic memory and spatial navigation has long been investigated in both animal and human research. In a seminal study, Edward C. Tolman [1] demonstrated that rats trained to navigate a specific path to a food goal in a sunburst maze could immediately select the correct novel alternative path when their original route was blocked. This finding shows that animals do not merely memorize rigid sequences of muscle movements (stimulus–response) but instead form an internal “cognitive map” of the environment that enables flexible problem-solving. Subsequent electrophysiological evidence from John O’Keefe and Dostrovsky [2] identified place cells in the hippocampus, further supporting the idea that the hippocampus plays a fundamental role in spatial representation and navigation [3]. These specialized neurons function as an internal positioning system, firing only when an animal occupies a specific location in its environment. Together, the collective firing of these cells forms a dynamic neural map that provides the physical substrate for Tolman’s conceptual cognitive map. Endel Tulving [4] introduced the concept of episodic memory, distinguishing it from semantic memory and emphasizing the difference between knowing and remembering—that is, between decontextualized knowledge and the conscious recollection of personally experienced events. Over the following decades, theoretical frameworks increasingly converged on the idea that episodic memory and spatial navigation share common neural and cognitive mechanisms. Several influential models have attempted to account for this overlap. Neuroimaging evidence further supports the overlap between networks involved in episodic memory retrieval and spatial navigation [5,6] in line with the hypothesis that memory and planning mechanisms may have evolved from neural systems originally developed for spatial navigation [7]. Moreover, emotional valence, that is, whether a stimulus is perceived as positive, negative or neutral, appears to modulate navigational memory: emotionally salient landmarks—material (pictures in this case) that can hold a pleasant or unpleasant value—have been shown to enhance recognition and long-term retention [8,9,10], although research in this domain remains relatively limited [11]. These studies aim to investigate the effect of emotionally laden stimuli on route navigation. A study by Ruotolo et al. [12] enriches this body of research, providing evidence on how participants retain basic information about landmarks.

That study further showed that the effect is valence-specific: participants who explored a virtual route containing positive images located those landmarks along the route more accurately than participants who were exposed to negative or neutral images.

Beyond valence (e.g., positive, negative, neutral), emotional stimuli also differ in arousal, which further affects memory performance. Many studies show that high-arousal items are better remembered than low-arousal items, thereby enhancing the individual’s memory by assigning a higher priority status to that information [13].

Chan and colleagues [14] tested participants performing an object location task in an fMRI study and found that people exposed to negative stimuli were more efficient in retrieving and positioning the stimulus as compared to participants exposed to positive stimuli.

This group of researchers hypothesized that remembering the position of negative stimuli could facilitate navigating an environment, allowing potential danger to be avoided; a proposal consistent with evidence that negative affect narrows processing towards local detail [15]. Most of the studies in this area do not study these processes incidentally, limiting the ecological validity of their findings. Investigating encoding under more naturalistic conditions may therefore provide a better approximation of everyday memory, although the experimental setting itself inevitably introduces some degree of noise. Taking part in an experiment is not a common experience: the laboratory setting, the presence of the experimenter and the awareness of being observed may partly reduce the ecological validity of the paradigm, even when encoding is incidental.

From a clinical perspective, navigational deficits and episodic memory decline are well documented in both normal aging and early Alzheimer’s disease [6]. However, differentiating physiological from pathological aging remains challenging, particularly in the preclinical stages. One possible explanation lies in the limited ecological validity and sensitivity of standard neuropsychological assessments, which often rely on intentional encoding and structured testing conditions. In everyday life, by contrast, most events and navigational experiences are acquired incidentally rather than intentionally. Crucially, this distinction between intentional and incidental acquisition aligns with evidence from amnesic patients, which indicates that implicit and incidental memory processes may remain relatively preserved despite severe impairments in explicit and intentional memory [16,17].

Furthermore, studies comparing incidental and intentional encoding in virtual environments suggest that incidental memory may be less affected by aging than intentional memory [18]. Importantly, early neuropathological changes in Alzheimer’s disease involve medial temporal structures implicated in both episodic memory and spatial navigation [19]. Topographical disorientation has therefore been proposed as a potential early marker of pathological aging [20], and visuospatial navigation deficits have been associated with the conversion from mild cognitive impairment to Alzheimer’s disease [6]. Accordingly, difficulties in remembering recently experienced events or recognizing familiar places are frequently among the earliest complaints reported by individuals who subsequently receive a diagnosis of subjective cognitive decline, mild cognitive impairment, or Alzheimer’s disease. Among the cognitive domains most vulnerable in both physiological and early pathological aging are episodic memory and visuospatial navigation, functions that critically depend on the integrity of medial temporal lobe structures, particularly the hippocampus.

To place these findings within the most recent literature, several studies published over the past five years have refined our understanding of how encoding instructions and emotional valence jointly shape spatial and source memory. Using an affective orienting task, Symeonidou and Kuhlmann [21] showed that emotion-enhanced source memory is contingent on the encoding instruction: an affective orienting task produced better source memory for both positive and negative sources than for neutral ones, whereas integrative instructions directing attention to item–source relationships overshadowed the emotional benefit, indicating that the instructional set interacts with valence rather than uniformly amplifying it. A meta-analysis by Pereira and colleagues [22] specified this interaction further: across 85 experiments, arousal enhanced source memory accuracy whereas valence per se impaired it, and the benefit of arousal was substantially reduced under intentional relative to incidental learning instructions, consistent with the proposal that effortful encoding equalises performance across emotional and neutral material. In the spatial domain, Ruotolo and colleagues showed that landmark valence and arousal differentially affect distance estimation and route drawing [23], and, more recently, proposed a stage-dependent model in which emotional valence differentially affects spatial memory during encoding, maintenance, and retrieval depending on whether representations are egocentric or allocentric [24]. Consistent with the idea that emotional effects are task-specific rather than global, Lanini-Maggi and colleagues [25] found that negatively valenced narrative cues improved recall of route directions, whereas both positive and negative cues impaired landmark sequence learning relative to neutral ones. Finally, Cianfanelli and colleagues [26] provide a mechanistic constraint of direct relevance here: the binding of emotional content to spatial location survived interference with central executive resources but was abolished by interference with the episodic buffer, indicating that emotion–location binding can proceed without controlled attention. Together, these recent contributions reinforce the rationale for examining how encoding instructions interact with emotional valence across both episodic and navigational domains, as undertaken in the present study.

Nevertheless, standardized clinical tools specifically assessing navigational memory remain limited. The present study aimed to develop and preliminarily test a novel task investigating the relationship between episodic memory and navigational memory under two contrasting encoding conditions: intentional and incidental encoding. The paradigm approximates everyday situations in which route information and concurrent events may be acquired either incidentally or intentionally. The task was designed to examine the extent to which encoding instructions modulate episodic and navigational memory in a sample of healthy young adults while providing the behavioral and effect-size estimates needed to design a subsequent, adequately powered study extending this paradigm to older adults and individuals at risk for pathological aging.

## 2. Materials and Methods

### 2.1. Participants

Sixty-three healthy college students (28 females), aged between 20 and 33 years (M = 25; SD = 2.97), took part in the study. Participants were assigned to two groups according to the order in which the two tasks were administered: Group AB (*n* = 34; 16 females) and group BA (*n* = 29; 12 females). The present study was conceived as a pilot investigation to obtain empirical estimates derived directly from the specific experimental procedures adopted in this protocol. The sample size was determined to align with established sample sizes in previous neuropsychological literature utilizing the short Computerized Ecological Navigational Battery paradigm and its variants [27,28]. These preliminary findings obtained from this pilot cohort will be used to calculate study-specific effect size estimates, which will inform the a priori determination of the minimum sample size required for a subsequent, adequately powered confirmatory study. Inclusion criteria required all participants to have no history of neurological or psychiatric disorders, or alcohol or drug abuse. All participants read and signed an informed consent form before enrollment. The study was designed in accordance with the principles of the Declaration of Helsinki and was approved by the Territorial Ethics Committee Lazio Area 5.

### 2.2. Procedure

The experimental procedure consisted of a computer-based test administered individually to each participant. The entire experiment lasted approximately one hour, depending on each participant’s reaction time. Sessions were conducted at the Department of Psychology, Sapienza University of Rome. An ecological computerized navigational battery, previously validated in several studies conducted by our research group [28], was adapted to assess episodic and navigational memory. The paradigm used first-person video recordings of urban routes, filmed by a professional videographer in a real-world city environment. The adaptation for the present study primarily consisted of adding nine pictures from the International Affective Picture System (IAPS) [29], selected for their valence and arousal ratings (see Table 1). Note that the differences in arousal are mostly due to neutral images being per se less arousing. Other noticeable differences depend on the difficulty in finding stimuli that could be paired from a visual perspective, rather than solely on the metrics. At each crossroad, along the route, one picture was presented as a concurrent episodic stimulus tied to a specific spatial decision point.

The order of picture presentation was fixed and was not randomized across participants or across repetitions, ensuring consistency with the landmarks and route progression. The video was presented three times. To assess incidental memory, two versions of the task were created. Two comparable routes from the short version of the navigational battery were used, along with two comparable sets of IAPS images. The two versions differed only in the instructions provided: in one version, participants were instructed to pay attention to the route, as they would later be asked questions about it (A); in the other version, participants were instructed to pay attention to the images, as they would later be asked questions regarding episodic material (B). The two versions were administered in counterbalanced order across participants (AB or BA).

**Figure 1:**
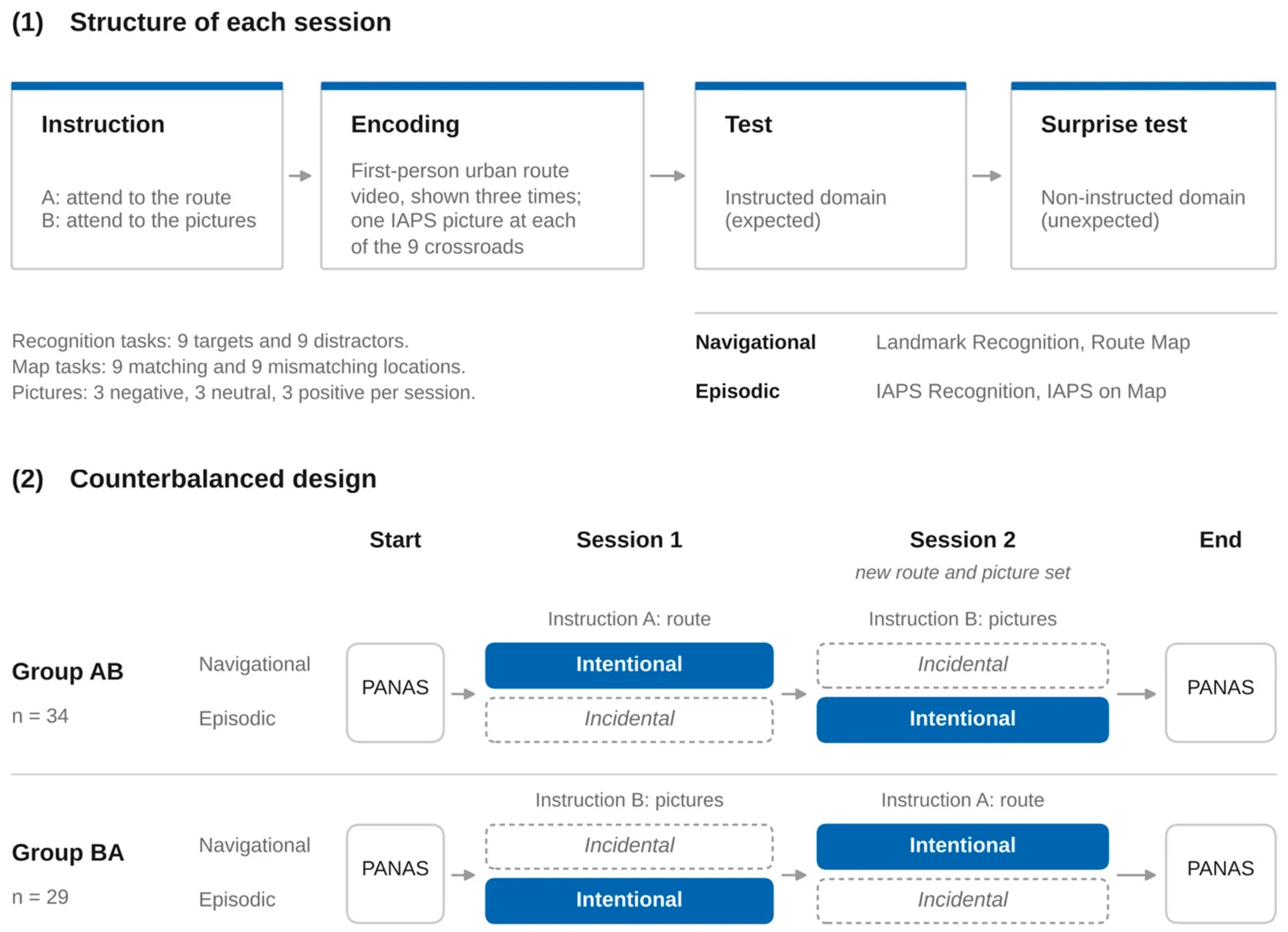
Experimental design. (**1**) Structure of each session: participants received instruction A (attend to the route) or B (attend to the pictures), watched the route video three times, and were then tested first on the instructed domain and then, unexpectedly, on the other domain. (**2**) Counterbalanced design: group AB received instruction A in Session 1 and instruction B in Session 2; group BA was the reverse. Filled boxes indicate intentional encoding and dashed boxes indicate incidental encoding. Affective state (PANAS) was assessed at the beginning and at the end of the experiment.

Contrary to the instructions provided, the recognition tests assessed memory for the entire material presented, regardless of which domain participants had been instructed to attend to. This design allowed the simultaneous assessment of intentional and incidental memory within the same experimental session. However, it should be noted that during the second session, participants were likely aware that both domains would be tested, given their experience with the full test battery in the first session. This awareness may have prompted more strategic encoding in the second session, partially attenuating the difference between intentional and incidental conditions. Figure 1 summarizes the design.

#### 2.2.1. Tasks

Following the video presentation, participants were administered various tests assessing the same abilities in episodic and navigational memory. Depending on the specific instruction condition received during the encoding phase (intentional navigation vs. intentional episodic), participants performed either the navigational or the episodic tasks first. The second task was then administered as a “surprise recognition task”, in which, unbeknownst to them, they were tested on material they had acquired incidentally.

Recognition and map tasks provided two behavioral measures: accuracy (total number of correct responses; range: 0–18) and mean reaction time (RT, in milliseconds). RTs were log-transformed before analyses. For the IAPS tasks, hit scores were also calculated separately for negative, neutral, and positive stimuli (range: 0–3 per valence category).

#### 2.2.2. Positive and Negative Affect Schedule (PANAS)

Because the pictures embedded in the navigational task had different emotional valence and arousal and given that participants’ emotional state could influence their memory performance [8], state affect was monitored. The emotional state of the participants was assessed using the Italian version of the Positive and Negative Affect Schedule (PANAS) [30,31] at both the beginning (pre-test) and the end (post-test) of the experiment. Scored on a 5-point Likert scale, ranging from 1 (very slightly or not at all) to 5 (extremely), the test consisted of 10 positive adjectives and 10 negative adjectives.

#### 2.2.3. Recognition Tasks

To assess landmark recognition, participants were presented with 18 photographs, one at a time, depicting 9 actual landmarks encountered along the path and 9 novel distractors. As an index of landmark knowledge, we calculated the total number of correct responses (max = 18) [28]. Additionally, an identical recognition task was performed using the episodic IAPS stimuli, consisting of 18 pictures (9 target images encountered along the route and 9 novel distractors; max = 18).

#### 2.2.4. Map Tasks

Each trial of this task displayed a screenshot of one of the crossroads encountered along the path in the upper-left corner, alongside the real map of the surrounding city area in the center of the screen. The entire path was indicated on the map with a red line, showing the starting and arrival points, and a placeholder marking the location of one of the crossroads. Participants had to indicate whether the presented crossroad in the upper-left corner corresponded to the placeholder’s position on the map by pressing the “s” key to say yes or the “n” key to say no. The task included 18 trials (9 matching and 9 mismatching configurations), and the total number of correct answers was computed and used as an index of survey knowledge (max = 18) [28]. To assess episodic structural layout memory, a parallel task configuration was also conducted with the IAPS images, in which the crossroad screenshots were replaced with concurrent episodic pictures (max = 18).

#### 2.2.5. Data Analyses

Analysis strategy: (i) A manipulation check examined whether affective state changed during the experiment. (ii) Linear mixed models were used as a secondary analysis to describe the full factorial structure (Domain × Session × Instruction Order), to test whether task order modulated the instruction effect, and to adjust for affective state. (iii) The primary analysis is the between-subjects comparison of the first session, which is free of carry-over from a previous session and of test expectancy. (iv) Signal detection analyses tested whether the primary effect reflected memory sensitivity rather than response bias. Additional analyses, including the separate analyses of groups AB and BA and the second-session comparison, are reported in the Supplementary Materials.

All data were analyzed in R (version 4.3.3) [32] using the tidyverse, lme4, lmerTest, emmeans, afex, and openxlsx packages. Most memory variables deviated from normality (Shapiro–Wilk tests, 42 of 60 tests *p* < 0.05); non-parametric tests were therefore used for all group comparisons. Effect sizes for non-parametric tests are reported as rank-biserial correlations; for paired comparisons, W is the sum of the ranks of the positive differences (intentional minus incidental). The significance threshold was set at α = 0.05 for all analyses.

As a manipulation check, and following Palmiero and Piccardi [8], changes in affective state from pre- to post-task were examined separately for positive and negative affect with a mixed ANOVA, with Time as a within-subject factor and Instruction Order as a between-subject factor; this parametric model was retained for comparability with [8]. Spearman rank-order correlations were computed between PANAS scores (pre-test, post-test, and change scores) and IAPS performance measures, with *p*-values Holm-corrected across the full set of correlations.

Linear mixed models (LMMs), chosen for their robustness to distributional violations with samples of this size, were fitted separately to recognition accuracy, recognition log reaction time, map accuracy and map log reaction time. Each model included Domain (episodic vs. navigational), Session (first vs. second) and Instruction Order (AB vs. BA) as fixed factors, with participant as a random intercept. Pre-test PANAS scores and pre-to-post change scores (post-test minus pre-test) for positive and negative affect were included as covariates, and their contribution was evaluated with likelihood ratio tests. Post hoc contrasts were computed with the emmeans package, using Bonferroni correction and Kenward–Roger degrees of freedom. These models were used to test whether task order modulated the effect of the instructions.

The main analysis compared the two groups on the first session with Mann–Whitney U tests. At that point neither group had yet experienced the surprise test, so this comparison is free of carry-over from a previous session and of test expectancy: for each domain, participants who had encoded it intentionally were compared with those who had encoded it incidentally. For the IAPS on map task, *p*-values for accuracy and for hits in each valence category were Holm-corrected.

Signal detection indices were computed to distinguish memory sensitivity from response bias. These analyses were restricted to the map tasks, because in the recognition tasks, hit rates were at ceiling and false-alarm rates at floor, leaving d′ undefined in practice. Hits were “yes” responses to matching trials and false alarms “yes” responses to mismatching trials. Sensitivity (d′) and response criterion (c) were computed with a log-linear correction [33], adding 0.5 to the number of hits and false alarms and 1 to the number of signal and noise trials. Indices were compared between instruction-order groups with Mann–Whitney tests, and the effect of valence was tested within each group with Friedman tests followed by Bonferroni-corrected pairwise comparisons.

The following analyses are reported in the Supplementary Materials. The between-subjects comparison was repeated for the second session, in which the encoding assignment was reversed, to assess whether the instruction effect persisted once both groups had experienced the task (Table S1). Wilcoxon signed-rank tests compared each participant’s intentional and incidental performance across the two sessions, separately for group AB and group BA (Tables S2 and S3).

## 3. Results

### 3.1. Affective State Manipulation Check

**Figure 2:**
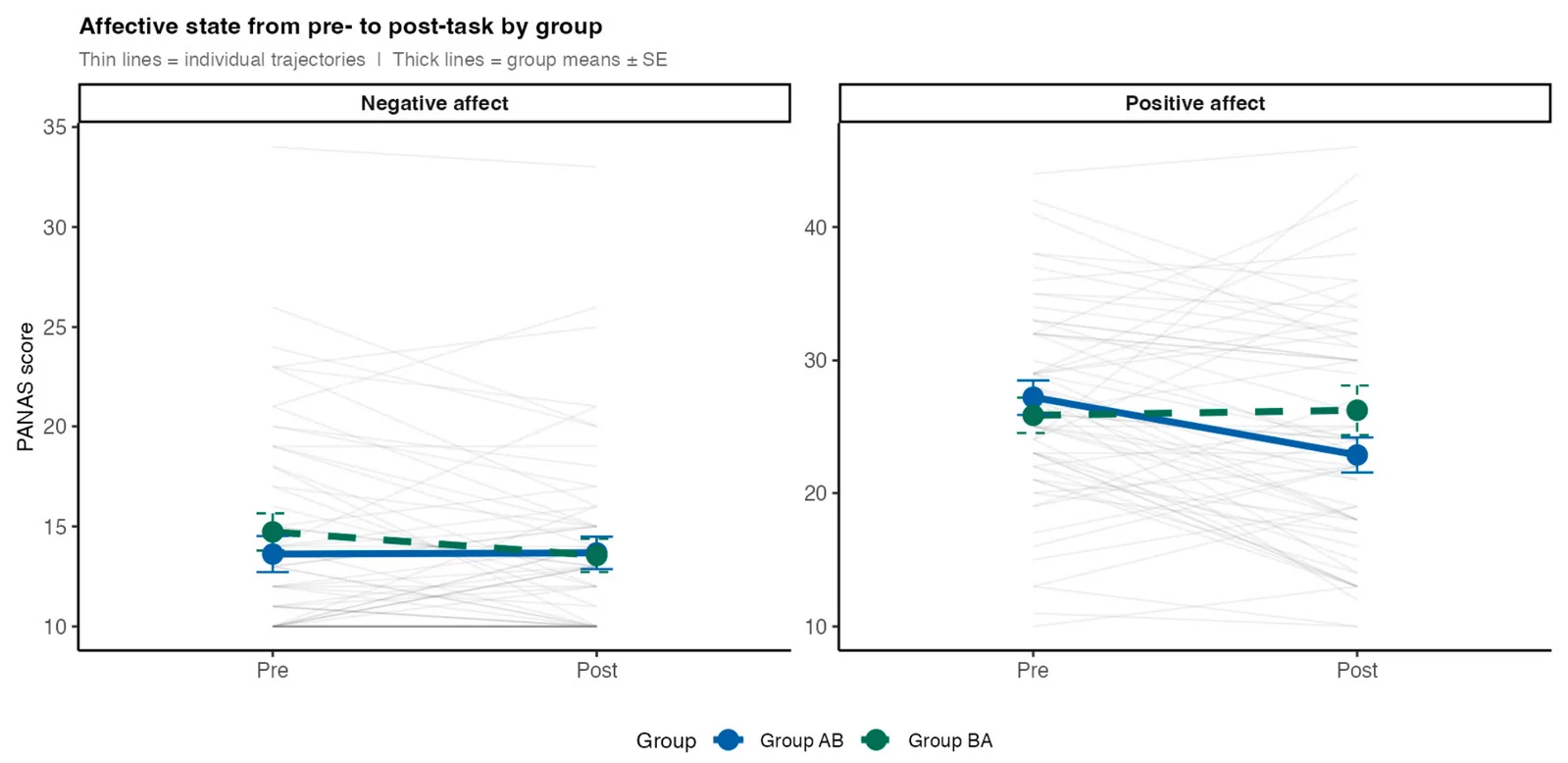
Affective state from pre- to post-task by group. Thin grey lines show individual trajectories; thick lines show group means ± SE.

Following Palmiero and Piccardi [8], we conducted a manipulation check to verify that performance differences were not attributable to mood changes. Changes in affective state were examined using a mixed ANOVA with Time (pre, post) as a within-subject factor and Order (AB, BA) as a between-subject factor, conducted separately for positive and negative affect (Figure 2). For positive affect, results revealed a significant main effect of Time, F(1, 61) = 6.05, *p* = 0.017, and a significant Time × Order interaction, F(1, 61) = 8.60, *p* = 0.005. In contrast, the main effect of Order was not significant, F(1, 61) = 0.28, *p* = 0.600. Post hoc comparisons showed that positive affect significantly decreased from pre- to post-task in Group AB (Mdiff = 4.32, *p* < 0.001). In contrast, no significant change emerged in Group BA (Mdiff = −0.38, *p* = 0.749). No significant between-group differences were found at either the pre-test or the post-test. For negative affect, neither the main effect of Time, F(1, 61) = 2.09, *p* = 0.153, nor the Time × Order interaction, F(1, 61) = 2.56, *p* = 0.115, reached significance.

To further clarify the nature of affective changes, separate paired-samples Wilcoxon signed-rank tests were conducted on the two PANAS subscales across the full sample. Results confirmed a significant decrease in positive affect from pre- to post-task (W = 580, *p* = 0.009, r = 0.387), with mean scores decreasing from 26.6 (SD = 7.41) to 24.4 (SD = 8.91), indicating a medium effect. In contrast, negative affect did not significantly change (W = 394, *p* = 0.238, r = 0.204). None of the Spearman correlations between the six PANAS measures and the IAPS performance indices remained significant after Holm correction (all adjusted *p*-values > 0.17).

### 3.2. Linear Mixed Models: Factorial Structure

**Figure 3:**
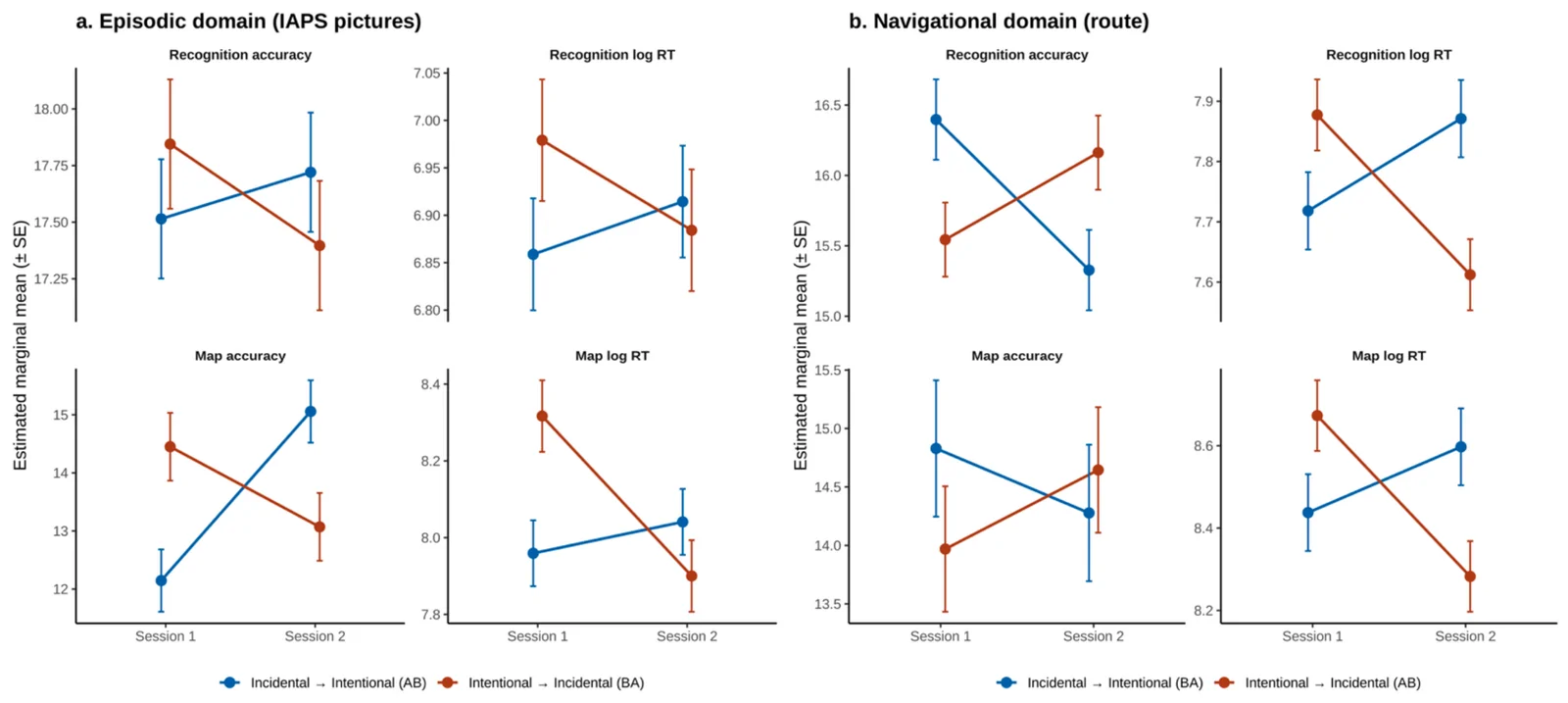
Estimated marginal means from the linear mixed models for the episodic (**a**) and navigational (**b**) domains, across the first and second sessions. In each domain, one group switched from incidental to intentional encoding and the other from intentional to incidental encoding (group AB: route attended first; group BA: pictures attended first). Error bars show standard errors.

To characterize the full factorial structure of encoding instruction effects, four LMMs were fitted (one per dependent variable: recognition accuracy and log reaction time, map accuracy and log reaction time), including Domain (episodic vs. navigational), Session (first vs. second) and Instruction Order (AB vs. BA) as fixed effects, along with four PANAS covariates. All models included a by-participant random intercept (Table 2, Figure 3).

The three-way interaction Domain × Session × Instruction Order was significant in three of the four models: map accuracy, F(1, 183) = 7.13, *p* = 0.008; map log RT, F(1, 183) = 36.58, *p* < 0.001; and recognition log RT, F(1, 183) = 22.60, *p* < 0.001. It did not reach significance for recognition accuracy, F(1, 183) = 2.29, *p* = 0.132. The Session × Instruction Order interaction was significant for map accuracy, F(1, 183) = 23.16, *p* < 0.001; recognition accuracy, F(1, 183) = 11.77, *p* < 0.001; and recognition log RT, F(1, 183) = 5.00, *p* = 0.027, but not for map log RT (*p* = 0.765). Instruction Order had no main effect and did not interact with Domain in any model (all *p* ≥ 0.43), indicating that performance did not differ overall between the two counterbalanced sequences. Domain was a significant main effect in all four models (all Fs > 6.8, all ps ≤ 0.01), reflecting overall differences in difficulty and scale between episodic and navigational tasks. Likelihood ratio tests showed that the four PANAS covariates as a block improved model fit for the two log RT models (recognition log RT, χ^2^(4) = 18.18, *p* = 0.001; map log RT, χ^2^(4) = 10.94, *p* = 0.027), but not for recognition accuracy (χ^2^(4) = 2.19, *p* = 0.701) and only marginally for map accuracy (χ^2^(4) = 7.83, *p* = 0.098). Within the map-accuracy model, however, the pre-to-post change in positive affect was significantly associated with overall map accuracy, F(1, 57) = 5.57, *p* = 0.022, although the PANAS covariates considered jointly did not significantly improve model fit. Post hoc contrasts (Bonferroni-corrected, Kenward–Roger degrees of freedom) examined group differences separately for each combination of Domain and Session. For the map accuracy model, a significant contrast emerged for the episodic domain in the first session (estimate = −2.304, SE = 0.811, t = −2.841, *p* = 0.005), with the intentional encoding group outperforming the incidental encoding group, and again in the second session with the opposite sign (estimate = +1.987, SE = 0.811, t = 2.449, *p* = 0.016), confirming the crossover pattern across instruction orders. No significant contrasts emerged for the navigational domain in either session (first session: *p* = 0.291; second session: *p* = 0.651), indicating that the instruction effect on map accuracy was specific to episodic memory and did not extend to navigational map performance.

The three-way interactions observed in the latency models followed a different, domain-crossed pattern. In the map log RT model, significant contrasts emerged for the episodic domain (IAPS on map) in the first session (estimate = −0.358, *p* = 0.007), indicating longer response latencies under intentional episodic encoding, and for the navigational domain (Route Map) in the second session (estimate = −0.315, *p* = 0.017). In the recognition log RT model, the only significant contrast involved the navigational domain (Landmark Recognition) in the second session (estimate = −0.259, *p* = 0.004). In the recognition accuracy model, both significant contrasts were navigational (Landmark Recognition) and of opposite sign across sessions (first session: estimate = −0.853, *p* = 0.032; second session: estimate = +0.834, *p* = 0.036), with no significant episodic contrast (both ps > 0.40). Taken together, these patterns indicate that the two domains were affected by the instruction manipulation in opposite directions across sessions. Consistently, when the two groups were analyzed separately (Supplementary Tables S2 and S3), intentional encoding improved IAPS on map accuracy in both Group AB (*p* < 0.001, r = 0.79) and Group BA (*p* = 0.021, r = 0.53), that is, regardless of whether intentional encoding took place in the first or the second session.

### 3.3. Between-Subjects Analysis

Mann–Whitney tests on the first-session conditions (Table 3) compared Group AB (*n* = 34; episodic incidental, navigational intentional) with Group BA (*n* = 29; episodic intentional, navigational incidental). Because neither group had yet been exposed to the full test battery, this comparison is uncontaminated by test expectancy.

**Figure 4:**
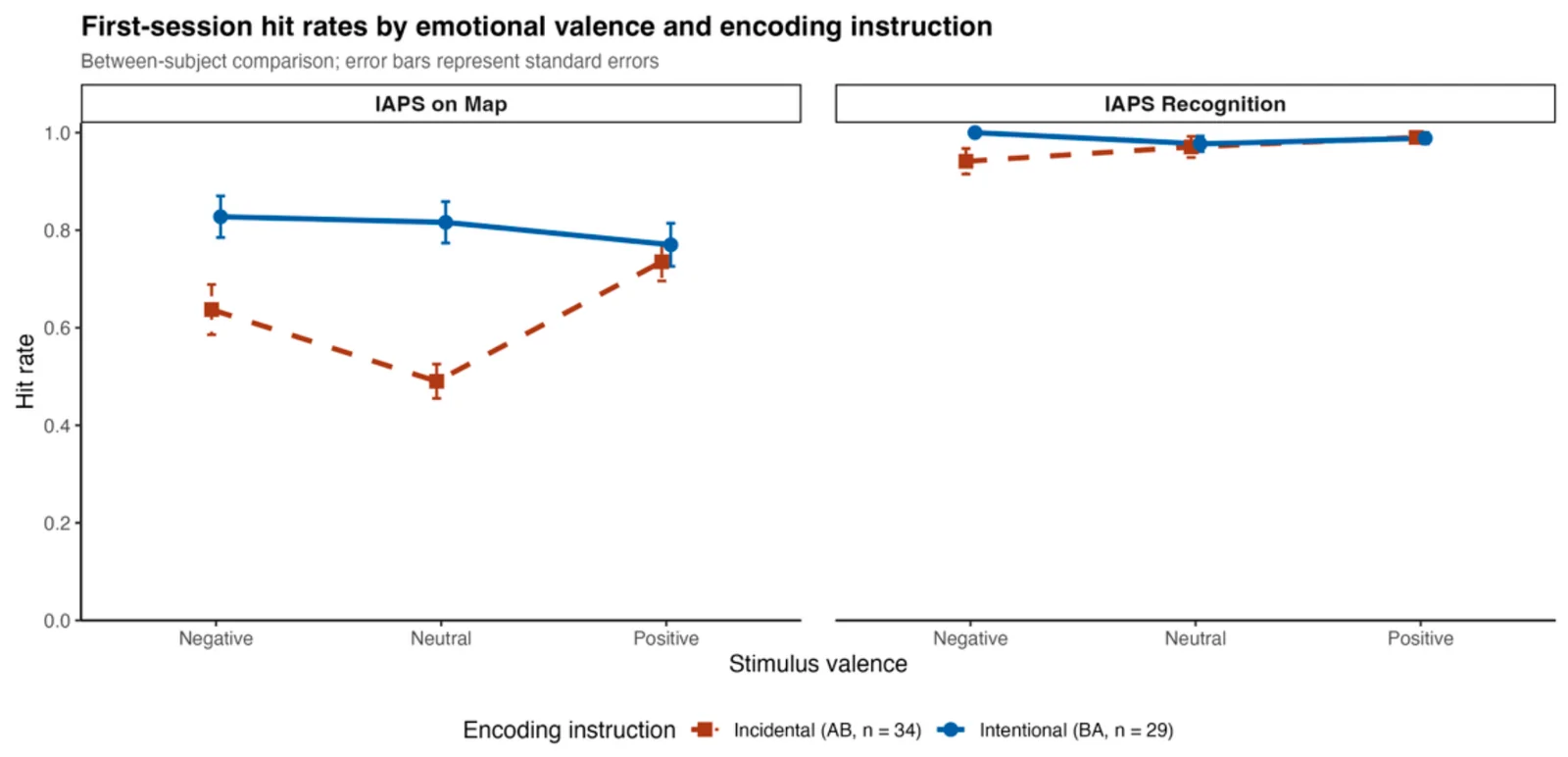
Hit rate by emotional valence and encoding instruction in the first session (incidental: group AB; intentional: group BA). Error bars show SE.

Significant intentional encoding advantages emerged for the IAPS on map task: accuracy (int: M = 14.690, SD = 2.929 vs. inc: M = 11.941, SD = 3.162; U = 259, *p* = 0.001, r = 0.475) and log RT (U = 281, *p* = 0.004, r = 0.430). Valence-specific IAPS on map HIT analyses showed significant advantages for negative (U = 316, *p* = 0.009, r = 0.359) and neutral stimuli (U = 165, *p* < 0.001, r = 0.665), but not for positive stimuli (U = 450, *p* = 0.522, r = 0.087; Figure 4).

After Holm correction across the four IAPS on map indices, the effects on accuracy (adjusted *p* = 0.004), neutral hits (adjusted *p* < 0.001) and negative hits (adjusted *p* = 0.018) remained significant, whereas positive hits did not differ (adjusted *p* = 0.522). For IAPS recognition, accuracy showed a small but significant difference (U = 349.5, *p* = 0.009, r = 0.291), as did log RT (U = 323, *p* = 0.019, r = 0.345); both were attenuated by the ceiling effects. After Holm correction across the four IAPS recognition indices, the accuracy effect remained significant (adjusted *p* = 0.036), whereas none of the valence-specific Recognition HIT indices differed between groups (negative: U = 420.5, *p* = 0.034, adjusted *p* = 0.102, r = 0.147; neutral: *p* = 0.909; positive: *p* = 0.928).

Critically, no significant group differences were observed for any navigational task in the first-session comparison (Landmark Recognition accuracy, *p* = 0.078; Landmark Recognition log RT, *p* = 0.366; Route Map accuracy, *p* = 0.104; Route Map log RT, *p* = 0.056), indicating that the instruction effect on first exposure was selective for episodic-emotional memory.

### 3.4. Signal Detection Analysis

**Figure 5:**
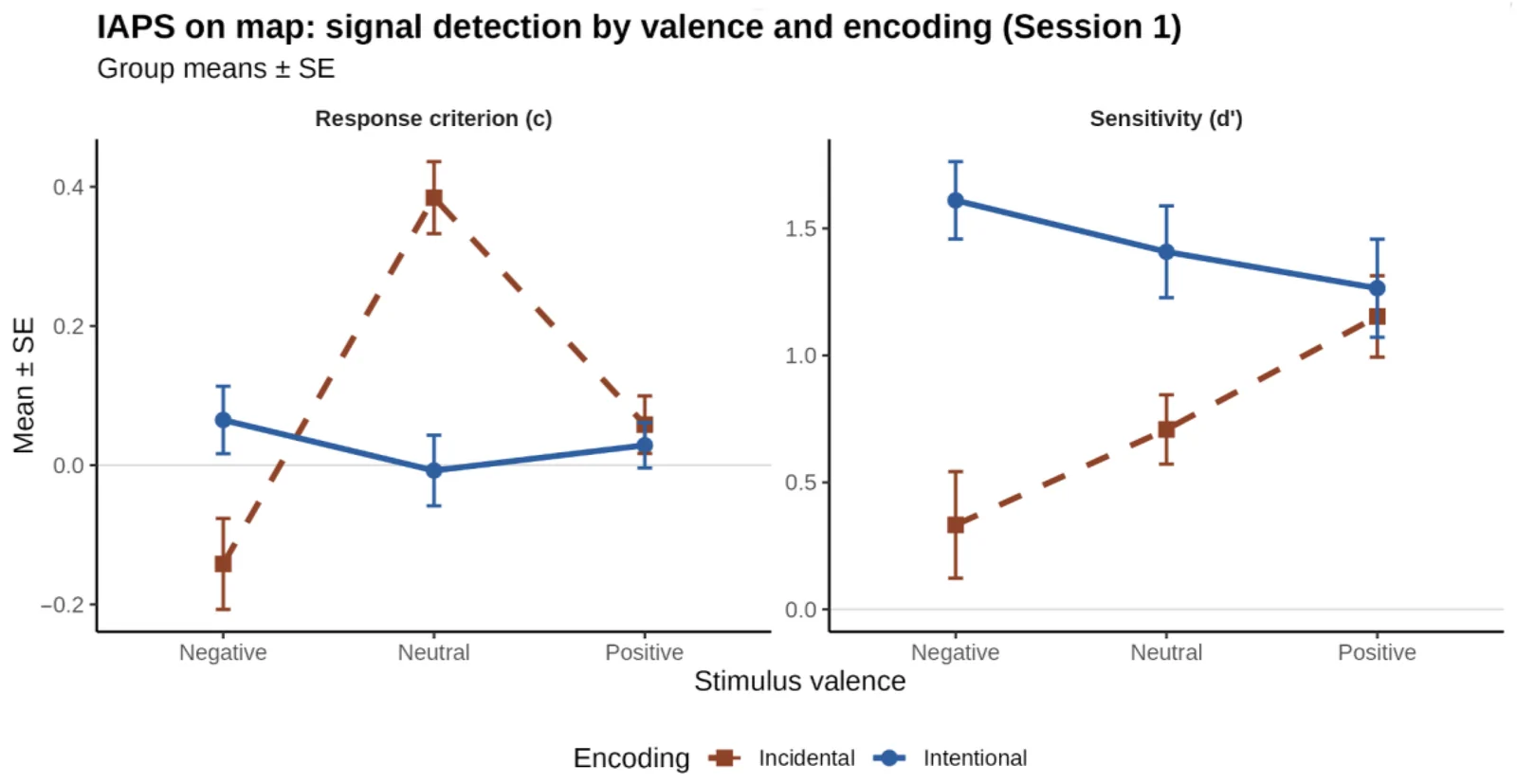
IAPS on map sensitivity (d′) and response criterion (c) by stimulus valence and encoding instruction in the first session (incidental: group AB; intentional: group BA). Points show group means ± SE.

Signal detection indices for the IAPS on map task were compared between the incidental group (AB, *n* = 34) and the intentional group (BA, *n* = 29) in the first session (Table 4). False-alarm rates in this task were substantial and variable (0.12–0.49), so d′ reflected memory discriminability rather than a hit-rate ceiling. Intentional encoding increased sensitivity for negative (d′ = 1.61 vs. 0.33; U = 200, *p* < 0.001, r = 0.594) and neutral pictures (1.41 vs. 0.71; U = 288, *p* = 0.004, r = 0.416), but not for positive pictures (1.27 vs. 1.15; U = 454.5, *p* = 0.587, r = 0.078) (Figure 5). The response criterion also differed between groups for negative (c = 0.07 vs. −0.14; U = 341, *p* = 0.021, r = 0.308) and neutral pictures (−0.01 vs. 0.38; U = 811, *p* < 0.001, r = 0.645), but not for positive pictures (U = 530, *p* = 0.534, r = 0.075).

**Figure 6:**
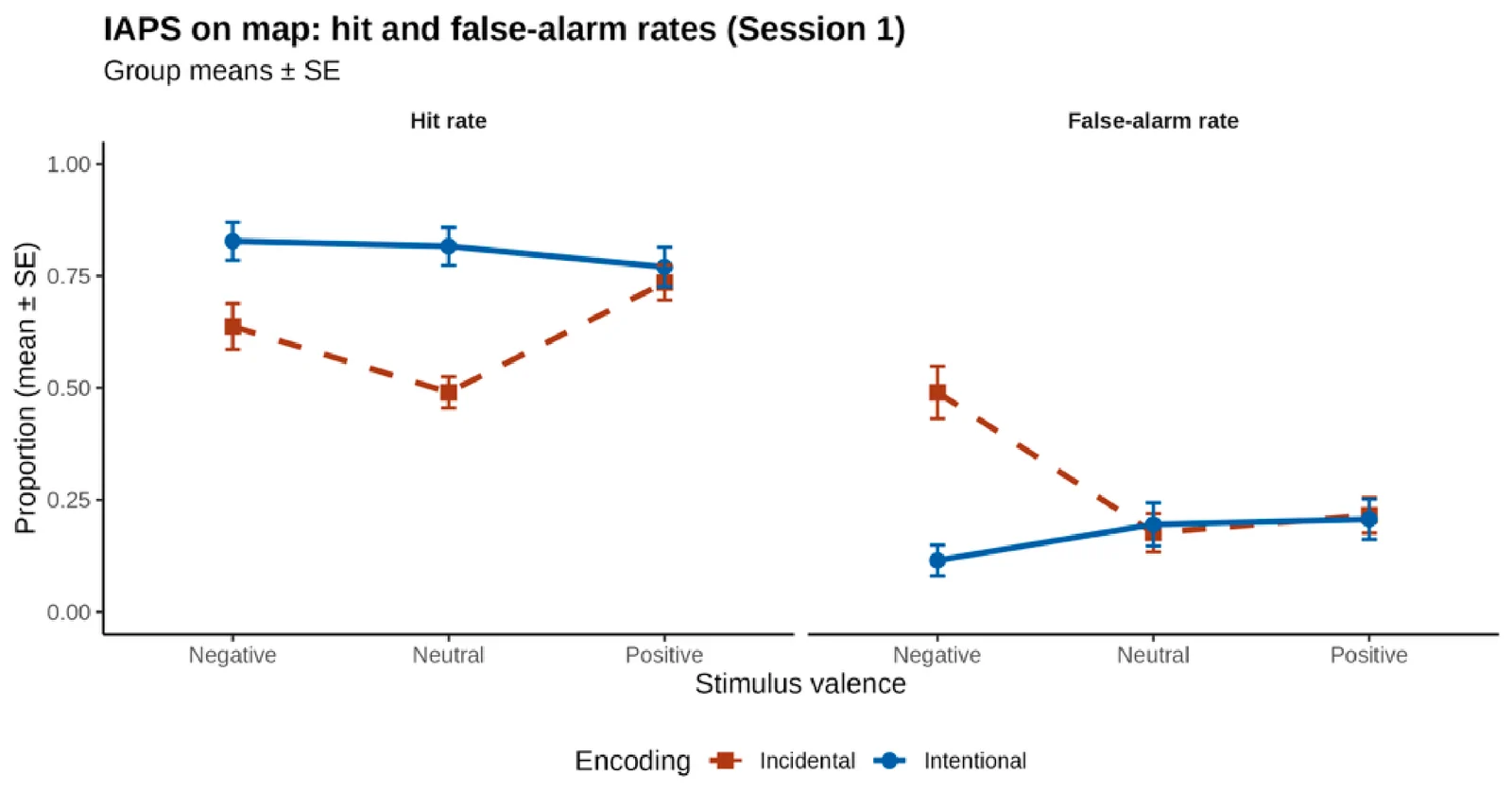
Hit and false-alarm rates for the IAPS on map task by emotional valence and encoding instruction in the first session (incidental: group AB; intentional: group BA). Error bars show SE.

The two criterion shifts went in opposite directions, more conservative for negative and more liberal for neutral pictures, so they cannot account for the parallel gain in sensitivity. In the incidental group, valence modulated both sensitivity (Friedman χ^2^(2) = 15.28, *p* < 0.001, Kendall’s W = 0.225) and the response criterion (χ^2^(2) = 23.21, *p* < 0.001, W = 0.341). Positive pictures were discriminated best (d′ = 1.15) and negative pictures worst (0.33; negative vs. positive, Bonferroni-adjusted *p* < 0.001, r = 0.865; neutral vs. positive, Bonferroni-adjusted *p* = 0.068, r = 0.513). In the intentional group, neither effect was significant (d′: χ^2^(2) = 3.63, *p* = 0.163; c: χ^2^(2) = 1.02, *p* = 0.602). Hit and false-alarm rates (Figure 6) show that intentional instructions acted differently on the two valence categories.

For negative pictures, intentional encoding reduced false alarms (0.12 vs. 0.49; U = 797.5, *p* < 0.001, r = 0.618) and increased hits (0.83 vs. 0.64; U = 316, *p* = 0.009, r = 0.359). For neutral pictures, the gain was carried by hits (0.82 vs. 0.49; U = 165, *p* < 0.001, r = 0.665), with no change in false alarms (0.20 vs. 0.18; *p* = 0.782). Neither rate differed for positive pictures (both ps > 0.52). Together, these results indicate that the intentional-encoding advantage for spatially bound emotional pictures reflects a gain in memory sensitivity that cannot be reduced to a change in response bias and that it is valence-selective: negative and neutral pictures benefited from intentional instructions, whereas positive pictures were already well discriminated under incidental encoding.

**Figure 7:**
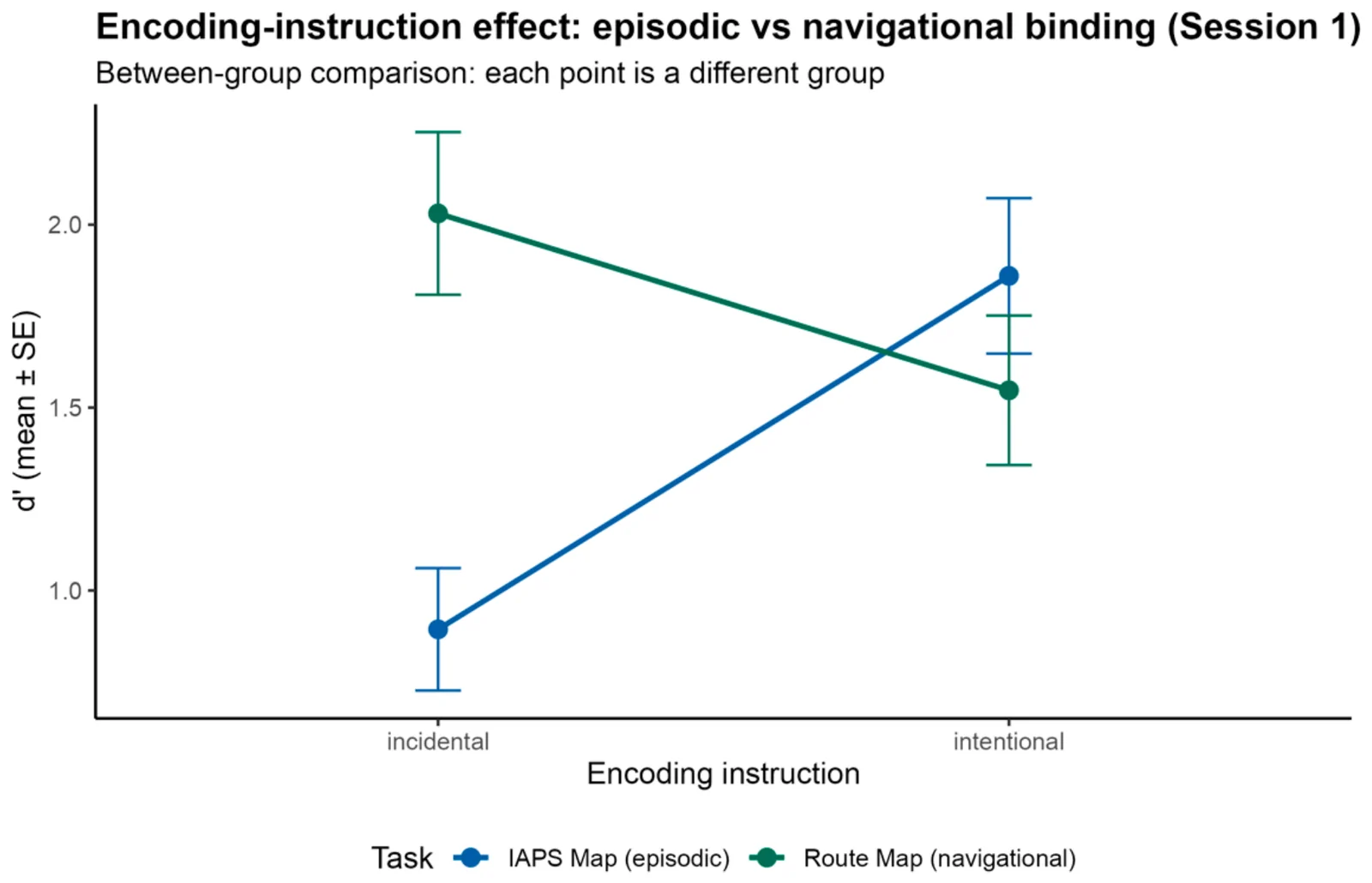
Sensitivity (d′) for the episodic IAPS on map task and the navigational Route Map task in the first session, by encoding instruction. Error bars show SE.

On the navigational Route Map task, scored on the same signal-detection scale (nine matching and nine mismatching configurations), no instruction effect emerged. Sensitivity was numerically lower in the intentional group (AB, d′ = 1.55) than in the incidental group (BA, 2.03; U = 379, *p* = 0.113, r = 0.231). By contrast, IAPS on map sensitivity computed over all nine targets and nine distractors was higher under intentional encoding (1.86 vs. 0.89; U = 271.5, *p* = 0.002, r = 0.449) (Table 5; Figure 7). This pattern was consistent with an instruction effect specific to the binding of emotional pictures to their locations, in line with the Domain × Session × Instruction Order interaction observed for map accuracy in the mixed models (Section 3.2).

## 4. Discussion

This study investigated whether intentional encoding instructions improve memory for emotional images and navigational information embedded in a first-person video, and whether affective state modulates these mnemonic processes. The manipulation check showed a decrease in positive affect from pre- to post-test, restricted to Group AB, whereas the two groups did not differ at the pre-test. None of the Spearman correlations between PANAS measures and IAPS performance remained significant after Holm correction. Although change in positive affect was a significant predictor in one mixed model, the PANAS covariate block did not significantly improve the overall fit of the map-accuracy model. Evidence for an association between affective change and memory performance was therefore limited and inconsistent across analyses. Moreover, because the PANAS was administered only before the first session and after the second, the timing of the observed change cannot be determined. Cognitive demands, emotional context and mood have previously been associated with memory performance [34,35,36,37]. However, the present design cannot determine whether these mechanisms contributed to the observed affective change; future studies should assess them directly.

The between-subjects analysis of the first session reported significant intentional encoding advantages for negative and neutral, but not positive, stimuli. The IAPS on map task was the most sensitive; the results show more accurate performance when the instructions explicitly state that attention should be paid to the images, revealing that binding an episodic picture to a spatial location requires explicit, top-down attentional resources. This is consistent with the view that associative source memory, particularly the binding of an object to its spatial context, depends on directed encoding rather than familiarity [38]. Zhou et al. [39] demonstrated using ERPs (Event-Related Potentials) that while familiarity-based processes (FN400) are sensitive to emotional valence, recollection-based source memory (LPC) is not modulated by valence, suggesting that spatial source binding relies on recollection and is therefore particularly sensitive to encoding depth. In the IAPS recognition task, the intentional condition numerically favored negative and neutral stimuli, whereas performance for positive stimuli was virtually identical across encoding conditions. Li et al. [40], using an fMRI paradigm with IAPS stimuli, confirmed that emotionally enhanced memory (EEM) is elicited by incidental encoding, particularly for positive high-arousal stimuli, with hippocampal activation correlated with recollection sensitivity. Only the comparison for negative stimuli reached statistical significance, although the effect size was small. Near-ceiling performance across valence categories likely limited the sensitivity of the task to detect additional instruction-related differences.

The most consistent finding of this study is the advantage of intentional over incidental encoding for episodic memory, particularly for the spatial reconstruction of emotional images (IAPS on Map). This effect emerged in the first-session comparison, which was unaffected by prior task exposure or surprise-test expectancy. It was also supported by the mixed models, in which instruction order had no main effect and did not interact with domain, and by the within-group comparisons, which showed the advantage in both Group AB and Group BA (Supplementary Tables S2 and S3), regardless of whether intentional encoding took place in the first or the second session. The valence selectivity observed in the signal-detection data further specifies this account. Under incidental encoding, sensitivity varied markedly across valence categories, with positive stimuli best discriminated and negative stimuli least; under intentional encoding this difference disappeared, as negative and neutral stimuli rose to the level of positive ones. Intentional instructions therefore appear to benefit precisely the material that is not already encoded spontaneously.

The signal-detection components further suggest that intentional instructions do not act in the same way on all valence categories. For negative images, the gain in sensitivity was driven mainly by a marked reduction in false alarms: once attention was directed to the pictures, participants stopped placing negative images at locations where they had not appeared. For neutral images, the gain was carried almost entirely by an increase in hits, with no change in false alarms, whereas for positive images neither component changed. This asymmetry suggests that negative images are spontaneously salient but poorly bound to a specific location, so that instruction mainly sharpens the location decision, whereas neutral images are neither salient nor bound and require instruction to be encoded.

A comparable pattern was reported by Ruotolo et al. [12], whose participants were asked to memorize a virtual route; positive landmarks were located more accurately along the route than negative or neutral ones. That instruction was not incidental, since memorization was explicitly requested; critically, however, it did not direct attention to the pictures or to their spatial position, and the resulting advantage for positive material parallels the pattern observed here whenever attention was not explicitly allocated to the images. The present findings extend this observation by manipulating the encoding instruction directly, and by showing that the advantage of positive material disappears once attention is explicitly directed to the pictures, as negative and neutral stimuli then rise to the same level.

The present data relate only in part to Knott et al. [41]: under incidental encoding, negative images produced the most false alarms, yet positive, not negative, images were best discriminated and gained least from instruction. In the present spatial paradigm, positive stimuli appear to achieve robust incidental encoding. Mather and Sutherland [13] emphasized that certain stimuli, through their arousal, are treated as high-priority information. Emotionally arousing stimuli draw attention to themselves, enabling a more precise binding of their details [42,43,44]. Palmiero and Piccardi [8] found that both positive and negative landmarks improved path learning relative to neutral ones, but only positive landmarks improved the reproduction of the path on a map, that is, the allocentric component of topographical memory. In the present study, positive images may function as affectively salient landmarks integrated into the spatial route representation, supporting allocentric encoding even without explicit instruction, consistent with evidence on emotional landmarks in spatial memory [9,45]. In the literature, positive stimuli are recalled more easily than negative stimuli that share the same arousal [46]. A body of research [40,47,48] indicates that positive emotions enhance memory performance across both general memory and heuristic aspects by intensifying the neural correlates of familiarity.

In the IAPS on map task, the results show that HIT values for positive stimuli do not decline when participants encode incidentally. Creating an allocentric representation of positive stimuli may reflect a smoother process that fosters a sense of familiarity. Iachini et al. [49] found that the locations of familiar buildings were typically represented in an allocentric frame of reference, whereas unfamiliar buildings were represented in an egocentric frame of reference, which could contribute to the observed differences in performance. Wang et al. [50], using ERPs, further demonstrated that familiarity-based recognition (FN400) is sensitive to emotional valence, providing a plausible neurophysiological mechanism for the robust incidental recognition of positive stimuli. Familiarity, as distinguished from recollection [51], plays a significant role throughout the battery, which was primarily designed to assess patients with neurodegenerative disease.

A second pattern concerns the navigational domain. Navigational performance did not benefit from intentional instructions: no group difference emerged on first exposure, and route sensitivity was, if anything, lower when the route was the declared target of attention, the opposite of what was observed for the spatial binding of emotional images. Emotional images placed at decision points may function as landmarks integrated into the route representation without requiring explicit attention to the path itself, so instructing participants to attend to the images did not impair spatial learning. This converges with Palmiero and Piccardi [8], who found that emotional landmarks enhanced path learning relative to neutral ones. This also argues against a general effort account: if intentional instructions simply mobilized more resources, performance should have improved in whichever domain was made the target of attention. Instead, only episodic performance benefited from being targeted, whereas route knowledge was acquired at least as well when attention was directed elsewhere. The instruction effect is therefore specific to the binding of emotional episodic content to spatial locations, consistent with evidence that route following and active wayfinding rely on partly distinct neural systems [52].

Even if the findings for episodic material are consistent not only with the literature but also across the different analyses conducted, the study has several limitations. First, the set of pictures is counterbalanced and chosen to be comparable; however, the objects depicted may carry different meanings for everyone. The set is standardized primarily on U.S. samples, and applying it to an Italian cohort could alter the emotional impact of stimuli. A review conducted by Branco and colleagues [53] proved the IAPS to be consistent across most populations considered, while also reporting some cross-cultural differences. The use of the original normative ratings in the present Italian cohort could be considered a potential limitation. To our knowledge, no comprehensive Italian normative standardization of the set is currently available; Balsamo et al. [54] conducted a bottom-up validation on Italian native speakers on a subset of pictures. Second, attention during encoding was not measured; although participants were tested individually in a laboratory setting, future studies should include an online measure of attention, such as eye tracking. Third, the PANAS was administered only at the beginning and at the end of the experiment, so we cannot establish when the decrease in positive affect occurred; an additional assessment between sessions should be included in future studies. Moreover, entering PANAS scores as covariates is a statistical adjustment that cannot fully exclude the influence of affective state on performance, particularly on response latencies, for which the PANAS covariates improved model fit (Section 3.2). Lastly, the analyses aim to address the ceiling effect encountered in recognition tasks; however, the experiment was designed to be administered to patients with neurodegenerative disease. Implementing active recall for both materials would remove the familiarity effect and possibly reduce the ceiling effect observed in young participants. Future directions will test patients using a refined paradigm to determine whether positive stimuli could be beneficial for treatment outcomes and rehabilitation programs.

## 5. Conclusions

This study provides preliminary evidence that intentional encoding instructions selectively enhance episodic–emotional memory, particularly the spatial binding of affective images, while leaving navigational memory unimproved. The valence-dependent pattern suggests that positive material benefits from automatic, familiarity-driven encoding processes that may be leveraged in clinical contexts. These findings are consistent with and extend a growing body of evidence demonstrating that the instruction-sensitivity of episodic encoding is jointly modulated by affective valence and spatial context [38,41,50]. Spatial navigation impairment has been identified as an early marker of neurodegenerative disease progression, particularly in amnestic mild cognitive impairment and Alzheimer’s disease [55], and hippocampal dysfunction underlying spatial learning deficits in aging has been linked to altered excitability [56]. Future research should test this paradigm in aging and patient populations to determine whether the instruction-independence of positive material encoding can be leveraged for early detection and cognitive rehabilitation.

## Figures and Tables

**Table 1:** Affective ratings (mean and standard deviation) of the target IAPS stimuli, presented by valence category. Values are based on the normative ratings reported in Lang et al. [29]. The 18 target pictures (6 per valence category) were divided into two sets of 9 (3 per valence), one for each version of the task. Ratings are on 9-point scales. Positive and negative pictures did not differ in arousal (*p* = 0.265), and both had higher mean arousal than neutral pictures.

Category	*n*	Valence M	Valence SD	Arousal M	Arousal SD
Negative	6	2.53	0.38	5.09	0.77
Neutral	6	5.45	0.45	3.81	0.42
Positive	6	7.57	0.56	4.62	0.59

**Table 2:** Linear mixed model fixed effects for the four dependent variables.

Effect	Recogn. acc. F	*p*	Recogn. RT F	*p*	Map acc. F	*p*	Map RT F	*p*
Domain	106.69	<0.001	828.80	<0.001	6.83	0.010	104.55	<0.001
Session	1.03	0.311	1.60	0.207	2.09	0.150	10.65	0.001
Instruction Order	0.00	0.981	0.43	0.517	0.10	0.753	0.49	0.488
Domain × Session	0.09	0.760	0.37	0.544	1.51	0.221	0.36	0.549
Domain × Order	0.00	0.986	0.01	0.936	0.02	0.879	0.63	0.429
Session × Order	11.77	<0.001	5.00	0.027	23.16	<0.001	0.09	0.765
Domain × Session × Order	2.29	0.132	22.60	<0.001	7.13	0.008	36.58	<0.001
PANAS positive pre	0.50	0.483	12.06	<0.001	1.97	0.166	7.12	0.010
PANAS negative pre	0.27	0.608	0.25	0.621	0.56	0.459	0.53	0.472
PANAS positive change	0.83	0.367	2.89	0.095	5.57	0.022	0.89	0.349
PANAS negative change	0.58	0.449	3.90	0.053	0.08	0.772	2.25	0.139

**Table 3:** Between-subjects comparisons of first-session conditions.

Task	Measure	Valence	*n* inc	M inc	SD inc	*n* int	M int	SD int	U	*p*	r
IAPS recognition	accuracy	collapsed	34	17.471	0.788	29	17.897	0.310	349.5	0.009	0.291
IAPS recognition	logRT	collapsed	34	6.836	0.235	29	7.005	0.291	323.0	0.019	0.345
IAPS recognition	HIT	negative	34	2.824	0.459	29	3.000	0.000	420.5	0.034	0.147
IAPS recognition	HIT	neutral	34	2.912	0.379	29	2.931	0.258	497.0	0.909	0.008
IAPS recognition	HIT	positive	34	2.971	0.171	29	2.966	0.186	495.5	0.928	0.005
IAPS on map	accuracy	collapsed	34	11.941	3.162	29	14.690	2.929	259.0	0.001	0.475
IAPS on map	logRT	collapsed	34	7.943	0.512	29	8.336	0.541	281.0	0.004	0.430
IAPS on map	HIT	negative	34	1.912	0.900	29	2.483	0.688	316.0	0.009	0.359
IAPS on map	HIT	neutral	34	1.471	0.615	29	2.448	0.686	165.0	<0.001	0.665
IAPS on map	HIT	positive	34	2.206	0.687	29	2.310	0.712	450.0	0.522	0.087
Landmark recognition	accuracy	collapsed	29	16.448	1.429	34	15.500	2.063	367.0	0.078	0.256
Landmark recognition	logRT	collapsed	29	7.744	0.388	34	7.855	0.479	559.0	0.366	0.134
Route map	accuracy	collapsed	29	15.069	3.116	34	13.765	3.376	376.0	0.104	0.237
Route map	logRT	collapsed	29	8.457	0.394	34	8.657	0.638	632.0	0.056	0.282

**Table 4:** Signal detection indices for the first session IAPS on map task, by stimulus valence and encoding instruction.

Valence	Encoding	d′ M (SD)	c M (SD)	Hit Rate	FA Rate
Negative	Incidental	0.33 (1.22)	−0.14 (0.38)	0.64	0.49
Negative	Intentional	1.61 (0.82)	0.07 (0.26)	0.83	0.12
Neutral	Incidental	0.71 (0.80)	0.38 (0.30)	0.49	0.18
Neutral	Intentional	1.41 (0.98)	−0.01 (0.27)	0.82	0.20
Positive	Incidental	1.15 (0.94)	0.06 (0.24)	0.74	0.22
Positive	Intentional	1.27 (1.04)	0.03 (0.18)	0.77	0.21

**Table 5:** Sensitivity (d′) for the first session episodic IAPS on map task versus the navigational Route Map task, by encoding instruction.

Task	Incidental d′	Intentional d′	Encoding *p*	r
IAPS on Map (episodic)	0.89 (0.98)	1.86 (1.14)	0.002	0.449
Route Map (navigational)	2.03 (1.20)	1.55 (1.19)	0.113	0.231

## Data Availability

The raw data supporting the conclusions of this article will be made available by the authors upon reasonable request.

## Supplementary Materials

The following supporting information can be downloaded at: https://www.mdpi.com/article/10.3390/brainsci16101113/s1, Table S1: Between-subjects comparisons of second-session encoding conditions; Table S2: Within-subjects comparisons of intentional versus incidental encoding in Group AB; Table S3: Within-subjects comparisons of intentional versus incidental encoding in Group BA.

## Abbreviations

The following abbreviations are used in this manuscript:

acc.	accuracy
ANOVA	analysis of variance
c	response criterion
d′	sensitivity index
FA	False Alarms
HIT	hit responses
IAPS	International Affective Picture System
LMM	Linear Mixed Model
M	mean
inc	incidental
int	intentional
PANAS	Positive And Negative Affect Schedule
RT	Reaction Time
SD	Standard Deviation
SE	Standard Error
SDT	Signal Detection Theory
