# Supplementary material for: Intentional Versus Incidental Encoding of Emotional and Navigational Memory: Preliminary Data in Healthy Young Adults
Source: Brain Sci. 2026 Oct 15;16(10):1113. doi: 10.3390/brainsci16101113 (PMC13702390; doi:10.3390/brainsci16101113)
Supplement: Supplementary file 1 [file brainsci-16-01113-s001.zip › brainsci-4590591-supplementary.pdf]

Supplementary Materials

Between-subjects analysis: second-session encoding conditions

Mann-Whitney tests on the second-session conditions (Table S1), in which the encoding assignment is reversed (Group AB now episodic intentional, Group BA episodic incidental), revealed a partially attenuated pattern in the episodic domain. For IAPS on map accuracy the intentional advantage persisted but was reduced in magnitude ($U = 661$, $p = .020$, $r = .341$). IAPS on map HIT analyses reached significance only for neutral stimuli ($U = 773$, $p < .001$, $r = .568$), with better performance in the intentional condition; negative ($p = .490$) and positive stimuli ($p = .677$) did not differ. IAPS Recognition accuracy was no longer significant ($p = .983$), consistent with the ceiling effects described in the main text, and IAPS on map log RT was attenuated to non-significance ($p = .273$). Among the valence-specific Recognition HIT indices, only neutral stimuli reached significance, with a negligible effect size ($U = 561$, $p = .028$, $r = .138$).

In contrast to the first session, several navigational differences emerged in the second session; all of them favored the incidental condition: Landmark Recognition accuracy was higher ($U = 636$, $p = .044$, $r = .290$) and both Landmark Recognition log RT ($U = 280$, $p = .003$, $r = .432$) and Route Map log RT ($U = 316$, $p = .015$, $r = .359$) were shorter for the group encoding the route incidentally. Route Map accuracy did not differ ($p = .862$). This attenuation of the episodic effect, together with the emergence of a reversed navigational effect, is consistent with the interpretation that the second session becomes de facto intentional through test expectancy, which compresses the difference between the two instruction conditions in the episodic domain while the attentional trade-off between domains becomes more visible.

Task	Measure	Valence	n inc	M inc	SD inc	n int	M int	SD int	U	*p*	r
IAPS recognition	accuracy	collapsed	29	17.448	1.920	34	17.676	0.806	491.5	.983	.003
IAPS recognition	logRT	collapsed	29	6.910	0.300	34	6.892	0.264	472.0	.777	.043
IAPS recognition	HIT	negative	29	2.862	0.441	34	2.912	0.379	514.5	.537	.044
IAPS recognition	HIT	neutral	29	2.793	0.620	34	3.000	0.000	561.0	.028	.138
IAPS recognition	HIT	positive	29	2.897	0.557	34	2.941	0.239	482.0	.695	.022
IAPS on map	accuracy	collapsed	29	13.310	2.867	34	14.853	3.135	661.0	.020	.341
IAPS on map	logRT	collapsed	29	7.920	0.418	34	8.024	0.458	573.0	.273	.162
IAPS on map	HIT	negative	29	2.207	0.819	34	2.353	0.734	539.5	.490	.094
IAPS on map	HIT	neutral	29	1.655	0.553	34	2.412	0.925	773.0	<.001	.568
IAPS on map	HIT	positive	29	2.241	0.912	34	2.353	0.812	521.0	.677	.057
Landmark recognition	accuracy	collapsed	34	16.118	1.903	29	15.379	1.741	636.0	.044	.290
Landmark recognition	logRT	collapsed	34	7.590	0.450	29	7.897	0.394	280.0	.003	.432
Route map	accuracy	collapsed	34	14.441	3.368	29	14.517	2.785	506.0	.862	.026
Route map	logRT	collapsed	34	8.266	0.529	29	8.617	0.487	316.0	.015	.359

Table S1. Between-subjects comparisons of second-session encoding conditions.

Within-subjects by group analysis

Wilcoxon signed-rank tests were used to compare each participant's intentional and incidental performance. Because the two encoding conditions were administered in counterbalanced order, results are reported separately for the two instruction-order groups.

AB Group

In Group AB (n = 34; Table S2), intentional encoding produced a large advantage for IAPS on map accuracy (W = 473, p < .001, r = .792). The valence-specific IAPS on map HIT analyses showed significant advantages for negative (W = 186, p = .009, r = .610) and neutral stimuli (W = 395, p < .001, r = .816), but not for positive ones (W = 140.5, p = .349, r = .216). IAPS Recognition measures showed no significant differences (all ps > .23), consistent with the ceiling effects described in the main text, nor did IAPS on map log RT (W = 344, p = .432). In the navigational domain, response latencies were significantly longer in the intentional condition for both Landmark Recognition (W = 507, p < .001, r = .704) and Route Map (W = 519, p < .001, r = .745), whereas accuracy did not differ significantly for either Landmark Recognition (W = 131, p = .099, r = .355) or Route Map (W = 171.5, p = .209, r = .262); in both cases the numerical direction slightly favored the incidental condition.

Task	Measure	Valence	n	M inc	SD inc	M int	SD int	M diff	SD diff	W	*p*	r
IAPS recognition	accuracy	collapsed	34	17.471	0.788	17.676	0.806	0.206	1.067	70.5	.257	.343
IAPS recognition	logRT	collapsed	34	6.836	0.235	6.892	0.264	0.056	0.209	367.0	.238	.234
IAPS recognition	HIT	negative	34	2.824	0.459	2.912	0.379	0.088	0.514	15.0	.374	.429
IAPS recognition	HIT	neutral	34	2.912	0.379	3.000	0.000	0.088	0.379	3.0	.371	1.000
IAPS recognition	HIT	positive	34	2.971	0.171	2.941	0.239	−0.029	0.300	2.0	.773	.333
IAPS on map	accuracy	collapsed	34	11.941	3.162	14.853	3.135	2.912	3.297	473.0	<.001	.792
IAPS on map	logRT	collapsed	34	7.943	0.512	8.024	0.458	0.082	0.470	344.0	.432	.156
IAPS on map	HIT	negative	34	1.912	0.900	2.353	0.734	0.441	0.894	186.0	.009	.610
IAPS on map	HIT	neutral	34	1.471	0.615	2.412	0.925	0.941	1.013	395.0	<.001	.816
IAPS on map	HIT	positive	34	2.206	0.687	2.353	0.812	0.147	0.892	140.5	.349	.216
Landmark recognition	accuracy	collapsed	34	16.118	1.903	15.500	2.063	−0.618	2.146	131.0	.099	.355
Landmark recognition	logRT	collapsed	34	7.590	0.450	7.855	0.479	0.265	0.385	507.0	<.001	.704

Task	Measure	Valence	n	M inc	SD inc	M int	SD int	M diff	SD diff	W	*p*	r
Route map	accuracy	collapsed	34	14.441	3.368	13.765	3.376	−0.676	3.254	171.5	.209	.262
Route map	logRT	collapsed	34	8.266	0.529	8.657	0.638	0.391	0.494	519.0	<.001	.745

Table S2. Within-subjects comparisons of intentional versus incidental encoding in Group AB.

BA Group

In Group BA (n = 29; Table S3), intentional encoding again produced an advantage for IAPS on map accuracy (W = 248, p = .021, r = .526), although the pattern was more selective than in Group AB. Significant effects also emerged for IAPS on map log RT (W = 394, p < .001, r = .811) and IAPS Recognition log RT (W = 316, p = .034, r = .453). The valence-specific IAPS on map HIT analyses showed a significant advantage only for neutral stimuli (W = 196, p < .001, r = .867), whereas negative (W = 68, p = .104, r = .495) and positive stimuli (W = 66.5, p = .723, r = .108) did not differ. As in Group AB, IAPS Recognition accuracy showed no significant difference (W = 16, p = .281), nor did any valence-specific Recognition HIT index (all ps > .17), all of which were again at ceiling.

In the navigational domain, Landmark Recognition accuracy was significantly higher in the incidental condition (W = 49.5, p = .002, r = .695), and response latencies were longer in the intentional condition (W = 353, p = .004, r = .623). Route Map accuracy did not differ significantly between conditions (W = 83, p = .261, r = .281), nor did Route Map log RT (W = 301, p = .073, r = .384).

Task	Measure	Valence	n	M inc	SD inc	M int	SD int	M diff	SD diff	W	*p*	r
IAPS recognition	accuracy	collapsed	29	17.448	1.920	17.897	0.310	0.448	1.956	16.0	.281	.524
IAPS recognition	logRT	collapsed	29	6.910	0.300	7.005	0.291	0.095	0.303	316.0	.034	.453
IAPS recognition	HIT	negative	29	2.862	0.441	3.000	0.000	0.138	0.441	6.0	.174	1.000
IAPS recognition	HIT	neutral	29	2.793	0.620	2.931	0.258	0.138	0.693	15.0	.374	.429
IAPS recognition	HIT	positive	29	2.897	0.557	2.966	0.186	0.069	0.593	2.0	1.000	.333
IAPS on map	accuracy	collapsed	29	13.310	2.867	14.690	2.929	1.379	2.981	248.0	.021	.526
IAPS on map	logRT	collapsed	29	7.920	0.418	8.336	0.541	0.417	0.438	394.0	<.001	.811
IAPS on map	HIT	negative	29	2.207	0.819	2.483	0.688	0.276	0.841	68.0	.104	.495
IAPS on map	HIT	neutral	29	1.655	0.553	2.448	0.686	0.793	0.902	196.0	<.001	.867
IAPS on map	HIT	positive	29	2.241	0.912	2.310	0.712	0.069	1.033	66.5	.723	.108
Landmark recognition	accuracy	collapsed	29	16.448	1.429	15.379	1.741	−1.069	1.557	49.5	.002	.695
Landmark recognition	logRT	collapsed	29	7.744	0.388	7.897	0.394	0.153	0.236	353.0	.004	.623
Route map	accuracy	collapsed	29	15.069	3.116	14.517	2.785	−0.552	2.585	83.0	.261	.281
Route map	logRT	collapsed	29	8.457	0.394	8.617	0.487	0.160	0.382	301.0	.073	.384

Table S3. Within-subjects comparisons of intentional versus incidental encoding in Group BA.
